# The Possibility of Suppression of Increased Postprandial Blood Glucose Levels by Gamma-Polyglutamic Acid-Rich Natto in the Early Phase after Eating: A Randomized Crossover Pilot Study

**DOI:** 10.3390/nu12040915

**Published:** 2020-03-27

**Authors:** Risa Araki, Keiko Fujie, Nanako Yuine, Yuta Watabe, Kazushi Maruo, Hiroaki Suzuki, Koichi Hashimoto

**Affiliations:** 1 Department of Clinical and Translational Research Methodology, Faculty of Medicine, University of Tsukuba, 1-1-1 Tennodai, Tsukuba, Ibaraki 305-8575, Japan; risa.araki@md.tsukuba.ac.jp (R.A.); k-fujie@md.tsukuba.ac.jp (K.F.); s1721330@s.tsukuba.ac.jp (N.Y.); s1721331@s.tsukuba.ac.jp (Y.W.); 2 Food Research Institute of National Agriculture and Food Research Organization, 2-1-12 Kannondai, Tsukuba, Ibaraki 305-8642, Japan; 3 Graduate School of Comprehensive Human Sciences, University of Tsukuba, 1-1-1 Tennodai, Tsukuba, Ibaraki 305-8575, Japan; 4 Department of Biostatistics, Faculty of Medicine, University of Tsukuba, 1-1-1 Tennodai, Tsukuba, Ibaraki 305-8575, Japan; maruo@md.tsukuba.ac.jp; 5 Department of Internal Medicine (Endocrinology and Metabolism), Faculty of Medicine, University of Tsukuba, 1-1-1 Tennodai, Tsukuba, Ibaraki 305-8575, Japan; hirosuzu@md.tsukuba.ac.jp

**Keywords:** gamma-polyglutamic acid, natto, postprandial glucose, insulin response, meal loading test, human health

## Abstract

The natto containing high levels of gamma-polyglutamic acid (γ-PGA) was recently developed. We investigated the effect of γ-PGA-rich natto consumption on postprandial glycemic excursion in humans. A randomized crossover meal test study was performed on healthy volunteers aged 20–64 years using the following test meals: (1) white rice (WR), (2) low-γ-PGA natto meal (WR + low-γ-PGA natto), and (3) high-γ-PGA natto meal (WR + high-γ-PGA natto). Blood samples were obtained at each visit before and for 120 min after loading. The incremental area under the curve (IAUC) of blood glucose and insulin levels was calculated and compared among the test meals. The blood glucose’s IAUC at 0–120 min, the primary endpoint, was 20.1% and 15.4% lower for the high- and low-γ-PGA natto meal than for the WR, with a significant difference only between the high-γ-PGA natto meal and WR (*p* < 0.05). The blood glucose’s IAUC at 0–15, 0–30, and 0–45 min was lower for the high-γ-PGA natto meal than for the low-γ-PGA natto meal (all *p* < 0.05). The possibility that high-γ-PGA natto might suppress blood glucose elevations in the early phase after eating is indicated.

## 1. Introduction

Japan has one of the longest life expectancies worldwide [1], and the traditional Japanese diet has attracted attention as a factor supporting the health and longevity of people in Japan [2]. Natto, the soybeans fermented by strains of *Bacillus subtilis*, is a frequently consumed traditional food in Japan [3], and the health benefits of its consumption, such as reduced risk of death from ischemic stroke [4] and improved bone strength [5], as well as reduced risk of hypertension with the consumption of natto and miso [6], have been suggested in epidemiological research. Further, gamma-polyglutamic acid (γ-PGA) [7], the main component of the viscous substance of natto, has been reported to improve lipid metabolism [8] and promote calcium absorption [9]. In addition, elevated blood glucose levels during an oral glucose tolerance test were found to be suppressed through the co-administration of γ-PGA potassium salt to C57BL/6J Jcl mice [10]. Karmaker et al. also reported the anti-diabetic effects of γ-PGA oxovanadium (IV) complex administration to KK-Ay mice [11]. The natto rich in γ-PGA is thought to have various health effects, and thus, the natto containing markedly higher γ-PGA levels (high-γ-PGA natto) was recently developed.

A study using a meal loading test for healthy subjects showed that the consumption of natto and naturally viscous vegetables with white rice (WR) reduces acute glycemia [12]. Ishikawa et al. suggested that natto is more useful than steamed soybeans for controlling postprandial glucose levels [13]. Thus, we performed meal loading tests to investigate whether the consumption of high-γ-PGA natto affects postprandial glycemic excursion, comparing it to the effects of low-γ-PGA natto or WR, and especially focused on the differences compared to low-γ-PGA natto. This is the first human intervention trial to evaluate the health benefits of high-γ-PGA natto.

## 2. Materials and Methods

### 2.1. Participants

Participants were recruited by the research staff via advertisements in local newspapers and the website of the University of Tsukuba. The inclusion criteria were as follows: age 20–64 years, body mass index (BMI) of 18.5–24.9 kg/m^2^, fasting plasma glucose (FPG) level < 126 mg/dL, hemoglobin A1c (HbA1c) level < 6.5%, and 2 h postprandial glucose levels after a 75 g oral glucose tolerance test < 200 mg/dL.

**Figure 1:**
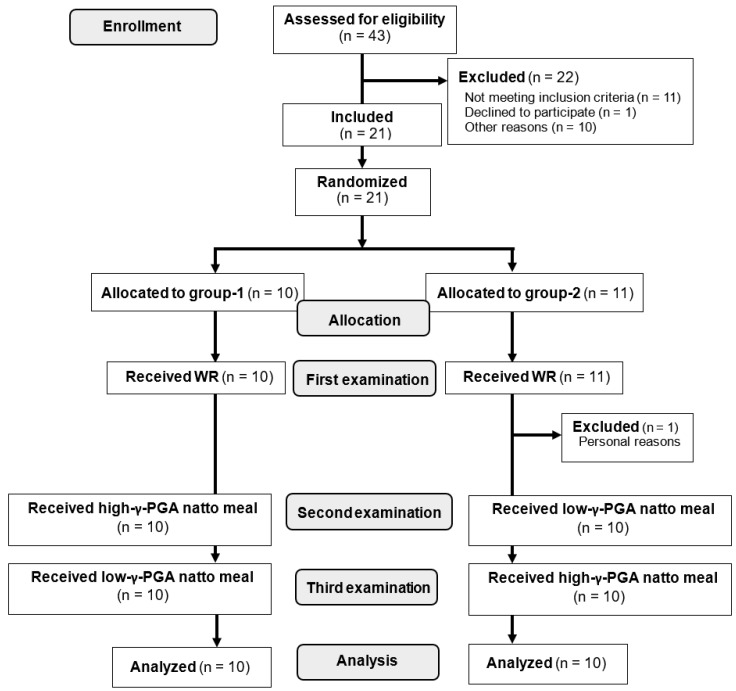
The diagram illustrating the selection of participants for analysis. WR: white rice; γ-PGA: gamma-polyglutamic acid.

Participants who had diabetes and/or anemia, were administered pharmacotherapy for any chronic disease, regularly consumed supplements (including the “foods for specified health use” and the “foods with function claims” approved by the Japan’s Consumer Affairs Agency) that affect glucose metabolism, and those who participated in other interventional studies were excluded from the study. Written informed consent was obtained from all the participants before study enrollment. Forty-three individuals were screened. Of the 22 eligible participants, one declined to participate, and 21 were enrolled in the study. The subjects were randomly assigned to either group 1 (n = 11) or group 2 (n = 10). One of the participants in group 2 completed the first examination, but declined to continue the study for personal reasons (Figure 1).

This study was performed by the research staff at the University of Tsukuba and was approved by the Clinical Research Ethics Committee of the University of Tsukuba Hospital (approval number: H29-207). The data were collected between February 1 and April 28, 2018, after the study was registered in the University Hospital Medical Information Network (UMIN) Clinical Trials Registry System (http://www.umin.ac.jp/ctr) as UMIN000030182. This study was conducted in accordance with the ethical principles expressed in the Declaration of Helsinki.

### 2.2. Study Protocol

The present study was a randomized controlled crossover pilot study. A meal loading test was conducted three times at intervals of at least 4 days. Baseline characteristics were measured to ensure study qualification. Each measurement was performed in the morning after fasting for ≥ 12 h. Blood was drawn from a vein before a test meal load and 15, 30, 45, 60, 90, and 120 min after test meal loading using an intravenous cannula, and blood glucose and insulin levels were measured. Meal loading tests were not performed during menstruation. The primary outcome measure was the incremental area under the curve (IAUC) of the blood glucose levels after consuming the test meal from the fasting state to the postprandial state at 120 min. The secondary outcome measures were the IAUC of the blood glucose and insulin levels at 0–15, 0–30, 0–45, 0–60, and 0–90 min, as well as the IAUC of the blood insulin levels at 0–120 min. These outcomes for the high-γ-PGA natto meal were compared to those of the other two test meals, especially focusing on the differences compared to the low-γ-PGA natto meal.

Three types of test meals were used as follows: (1) WR (150 g of packaged rice containing 48.5 g of carbohydrates), (2) high-γ-PGA natto meal (WR + 40 g of high-γ-PGA natto), and (3) low-γ-PGA natto meal (WR + 40 g of low-γ-PGA natto). All the participants were given WR at the first examination. We then assigned participants to receive both the high-γ-PGA natto meal and the low-γ-PGA natto meal in random order, with an allocation table using computer-generated random numbers prepared by a data coordinator based on a simple randomization method. The allocation sequence was concealed from the research staff other than the data coordinator until the interventions were assigned. At the second examination, group 1 was given the high-γ-PGA natto meal, whereas group 2 received the low-γ-PGA natto meal. At the third examination, each group received the test meal containing a natto preparation different from that of the second examination (Figure 1).

The participants were blinded to the meal allocation. Each meal was provided with 150 mL of water and consumed in 10 min. The composition and components of each meal are shown in Table 1. Packaged rice manufactured by Sato Foods Co., Ltd. (Niigata City, Niigata, Japan), and natto manufactured by the Natto Research Center of Takano Foods Co., Ltd. (Omitama City, Ibaraki, Japan), were used. Compared to the mean amount of γ-PGA in commercial natto [14], that of the low-γ-PGA natto was approximately one quarter, whereas that of the high-γ-PGA *natto* was approximately two times higher. The γ-PGA in both low- and high-γ-PGA natto was derived naturally, and the differences in γ-PGA amounts between the two types of natto were dependent on the types of *Bacillus subtilis* used for each fermentation.

### 2.3. Clinical Measurements

Body weight and height were measured with the participants wearing light clothing and no shoes. BMI was calculated as body weight in kilograms divided by height in meters squared (kg/m^2^). These data were collected at the baseline.

### 2.4. Analysis of Blood Biochemical Parameters

Blood glucose levels were measured using the hexokinase/glucose-6-phosphate dehydrogenase method. HbA1c was determined using the latex coagulation method and was expressed using the National Glycohemoglobin Standardization Program scale. Blood glucose and HbA1c were analyzed using a JCA-BM9130 analyzer (Japan Electron Optics Laboratory, Tokyo, Japan). Insulin levels were measured via chemiluminescence immunoassays using the Roche Modular Analytics E170 assay (Roche Diagnostics GmbH, Mannheim, Germany). The homeostasis model assessment of insulin resistance was calculated as fasting insulin (μU/mL) × FPG (mg/dL)/405. All laboratory examinations were performed at Kotobiken Medical Laboratories, Inc. (Tsukuba, Ibaraki, Japan).

### 2.5. Questionnaire Survey

The frequency of usual natto intake was investigated using a questionnaire with five alternatives (every day, almost every day, 3–4 times per week, 1–2 times per week, less than once per week) at the baseline. At the second and third examination, sensory evaluation of the low- and high-γ-PGA natto was performed. Particularly, the degree of perceived “hardness,” “viscosity,” and “flavor” of the test meal compared to the natto that the subjects typically consumed was evaluated and scored from 1 to 5 (lower: 1 point, slightly lower: 2 points, equal: 3 points, slightly higher: 4 points, higher: 5 points).

### 2.6. Statistical Analysis

A paired *t*-test was used to compare the scores of sensory evaluations between low- and high-γ-PGA natto. Each IAUC of the blood glucose and insulin levels after consuming the test meal from the fasting state to the postprandial states at 15, 30, 45, 60, 90, and 120 min was calculated using the trapezoidal rule, and these data were used for analysis. The primary endpoint was blood glucose’s IAUC 0–120 min. For the two participants who deviated from the protocol on one of the three measurement days, the blood glucose levels at all measurement points on that day were treated as missing data. A linear mixed model analysis was used to compare blood glucose’s IAUC between the test meals, with the blood glucose’s IAUC at each point treated as a dependent variable, subjects as a random effect, and test meals and groups as fixed effects. A linear mixed model analysis was also performed to determine the blood insulin’s IAUC. The estimated mean ± the standard error (est. mean ± SE) was shown. For other descriptive statistics, the mean ± the standard deviation (mean ± SD) or the mean ± the SE was shown. All statistical analyses were performed using SPSS Statistics 22 (SPSS, Inc., Chicago, IL, USA), and a two-tailed *p* value of < 0.05 was considered significant.

## 3. Results

### 3.1. Baseline Characteristics

The twenty participants (group 1: n = 11, group 2: n = 9) who completed all three meal loading tests were evaluated. At the time of enrollment, the participants’ age ranged from 20 to 63 years, and their BMI ranged from 18.8 to 24.4 kg/m^2^. Three out of 20 individuals (15.0%) met the criteria for prediabetes defined by the American Diabetes Association (FPG 100–125 mg/dL and/or HbA1c 5.7–6.4%), whereas 17 subjects (85.0%) had normal FPG and HbA1c levels. For the average consumption frequency of natto, “1–2 times per week” was the most common answer (Table 2).

### 3.2. Sensory Evaluations of Natto Used in the Present Study

**Figure 2:**
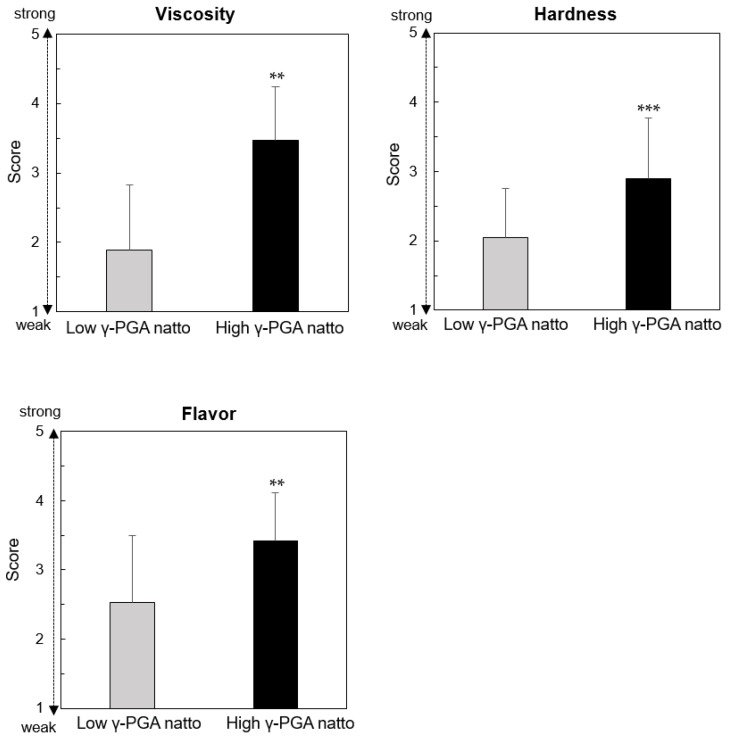
Sensory evaluations of the natto used in the present study. “Viscosity,” “hardness,” and “flavor” of high- and low-γ-PGA natto were compared using a paired *t*-test, and the data are shown as the mean ± the standard deviation (mean ± SD). ** *p* < 0.01, *** *p* < 0.001 vs. low-γ-PGA natto. γ-PGA: gamma-polyglutamic acid.

The scores for “viscosity,” “hardness,” and “flavor” of the high-γ-PGA natto were 3.5 ± 0.8, 2.9 ± 0.9, and 3.4 ± 0.7 points, respectively; they were significantly higher than those of the low-γ-PGA natto (1.9 ± 0.9 points, *p* < 0.01, 2.1 ± 0.7 points, *p* < 0.001, and 2.5 ± 1.0 points, *p* < 0.01; Figure 2).

### 3.3. Comparison of Blood Glucose’s IAUC between the Test Meals at Each Time Point

**Figure 3:**
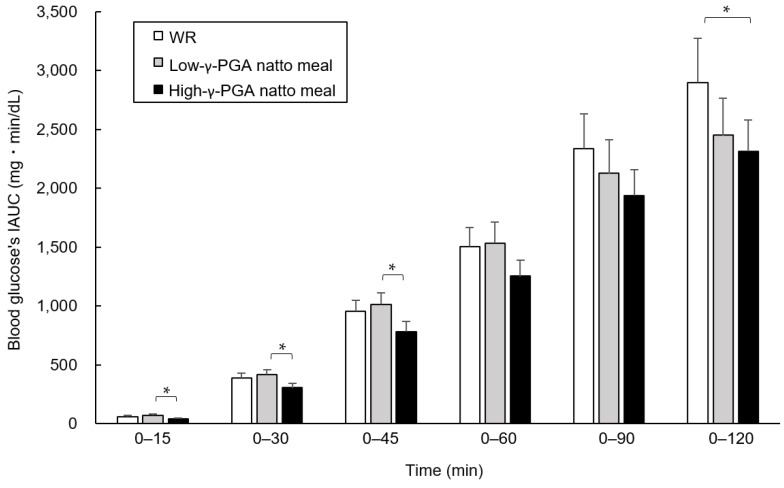
Comparison of the blood glucose’s incremental area under the curve (IAUC) between the test meals at each time point. The blood glucose’s IAUCs at 0–15, 0–30, 0–45, 0–60, 0–90, and 0–120 min were compared using a linear mixed model regression analysis with the test meals and groups as fixed effects, and the data are shown as the estimated mean ± the standard error (est. mean ± SE). * *p* < 0.05. WR: white rice; γ-PGA: gamma-polyglutamic acid.

The blood glucose’s IAUCs 0–120 min after the test meal loading showed no significant difference between the three test meals, except that the high-γ-PGA natto meal showed a value significantly lower than that of the WR (*p* < 0.05). The IAUC was in the order of WR (2895.8 ± 376.7 (est. mean ± SE) mg·min/dL), the low-γ-PGA natto meal (2450.2 ± 316.1 mg·min/dL), and the high-γ-PGA natto meal (2313.5 ± 265.7 mg·min/dL). The blood glucose’s IAUC of the high-γ-PGA natto meal was the lowest among the three test meals not only at 0–120 min, but also at other time points. Further, the blood glucose’s IAUC values of the high-γ-PGA natto meal at 0–15, 0–30, 0–45, and 0–60 min were significantly lower or tended to be lower than those of the low-γ-PGA natto meal (0–15, 0–30, and 0–45 min: *p* < 0.05, 0–60 min: *p* = 0.072) and WR (0–15 min: *p* = 0.051, 0–30 min: *p* = 0.072, 0–60 min: *p* = 0.091, 0–90 min: *p* = 0.070) (Figure 3).

### 3.4. Changes in Blood Glucose Levels Over Time Following Each Test Meal Loading

**Figure 4:**
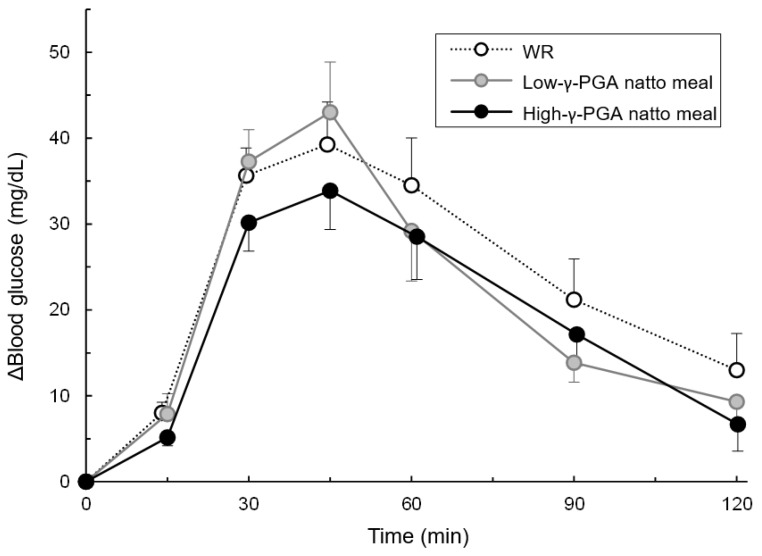
Changes in blood glucose levels over time following each test meal loading. The blood glucose elevation data from fasting to 15, 30, 45, 60, 90, and 120 min are shown as the mean ± the standard error (SE). WR: white rice; γ-PGA: gamma-polyglutamic acid.

As shown in Figure 4, peak blood glucose was reached at 45 min with all the test meals. The elevations in blood glucose levels from 0 to 45 min was in the order of the low-γ-PGA natto meal (43.0 ± 5.8 (mean ± SE) mg/dL), WR (39.3 ± 4.9 mg /dL), and the high-γ-PGA natto meal (33.9 ± 4.5 mg /dL).

### 3.5. Comparison of blood insulin’s IAUC between Test Meals at Each Time Point

**Figure 5:**
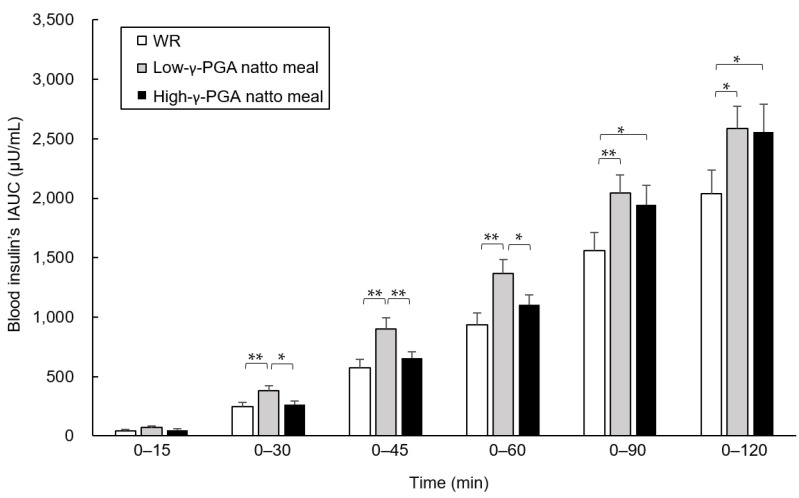
Comparison of the blood insulin’s incremental area under the curve (IAUC) between the test meals at each time point. The blood insulin’s IAUC at 0–15, 0–30, 0–45, 0–60, 0–90, and 0–120 min were compared using a linear mixed model regression analysis with the test meals and groups as fixed effects, and the data are shown as the estimated mean ± the standard error (est. mean ± SE). * *p* < 0.05, ** *p* < 0.01. WR: white rice; γ-PGA: gamma-polyglutamic acid.

The blood insulin’s IAUC of the high-γ-PGA natto meal was lower at 0–30, 0–45, and 0–60 min compared to that of the low-γ-PGA natto meal (0–30 and 0–60 min: *p* < 0.05, 0–45 min: *p* < 0.01). Compared to that of WR, the blood insulin’s IAUC of the low-γ-PGA natto meal was higher or tended to be higher at all time points (0–15 min: *p* = 0.050, 0–120 min: *p* < 0.05, 0–30, 0–45, 0–60, and 0–90 min: *p* < 0.01), whereas that of the high-γ-PGA natto meal was higher only at 0–90 and 0–120 min (both *p* < 0.05; Figure 5).

### 3.6. Changes in Blood Insulin Levels Over Time Following Each Test Meal Loading

**Figure 6:**
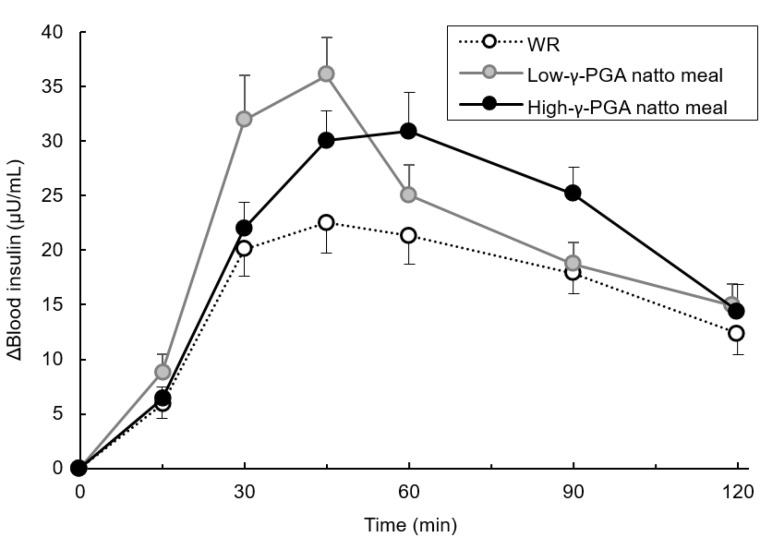
Changes in blood insulin levels over time following each test meal loading. The blood insulin elevation data from fasting to 15, 30, 45, 60, 90, and 120 min are shown as the mean ± the standard error (SE). WR: white rice; γ-PGA: gamma-polyglutamic acid.

As shown in Figure 6, a delayed peak in insulin secretion with the high-γ-PGA natto meal compared to that with the other two test meals was observed. The elevations in insulin levels from fasting to the peak point was in order of the low-γ-PGA natto meal (36.0 ± 2.8 (mean ± SE) mg /dL), high-γ-PGA natto meal (30.9 ± 3.6 mg /dL), and WR (22.5 ± 2.5 mg /dL).

## 4. Discussion

This study was conducted to examine the effect of consumption of high-γ-PGA natto on postprandial glycemic excursion in humans. The mean blood glucose’s IAUC at 0–120 min was 20.1% and 15.4% lower for the high-γ-PGA natto meal and the low-γ-PGA natto meal than for WR, and the blood glucose’s IAUC at 0–120 min was significantly different between the high-γ-PGA natto meal and WR. Compared to the blood glucose’s IAUC of WR at time points other than 0–120 min, that of the high-γ-PGA natto meal tended to be lower at all the time points except for at 0–45 min, whereas that of the low-γ-PGA natto meal showed no difference or trends at any time points. Furthermore, a comparison of the blood glucose’s IAUC of low-γ-PGA natto and high-γ-PGA natto suggested that high-γ-PGA natto was more effective in suppressing increases in blood glucose levels than low-γ-PGA natto and that the difference was large, particularly within 1 h after the meal.

Soybeans, the primary ingredient in natto, contain water-soluble fiber, soy protein, and isoflavone, and water-soluble fiber has been reported to slow down digestion and absorption and prolong the gastric retention time [15,16]. Taniguchi et al. observed that consumption of natto and naturally viscous vegetables with WR reduces acute glycemia in healthy individuals [12] and obese individuals with impaired glucose tolerance [17]. These benefits were attributed to the presence of a water-soluble fiber.

Ishikawa et al. conducted a meal loading test for healthy adult males and reported that natto is more useful for controlling postprandial glucose levels than steamed soybeans because of not only the water-soluble fiber derived from soybeans, the ingredient in natto, but also of the high viscosity of natto [13]. The high-γ-PGA natto used in this study contained approximately eight times more γ-PGA than the low-γ-PGA natto and was highly viscous. β-glucan is a viscous substance like γ-PGA, and it has been reported that as the viscosity of β-glucan is increased, the starch digestibility is reduced, and glucose responses are lowered [14]. In addition, a hyperglycemia inhibitor that consists primarily of the γ-PGA potassium salt has been patented [10]. Tamura et al. also reported that co-administration of γ-PGA suppresses the initial rise in blood glucose levels after starch loading in mice [18]. Therefore, the high-γ-PGA natto-induced suppression of blood glucose elevation in this study might have been affected by γ-PGA, the content of which was markedly different between the high- and low-γ-PGA natto meals.

The mean blood insulin’s IAUC of the low γ-PGA natto meal at all time points after 0–30 min and of the high-γ-PGA natto meal at 0–90 and 0–120 min was higher than that of the WR. Amino acids have been reported to promote insulin secretion in pancreatic β-cells [19]; in this study, it was thought that insulin secretion was increased by simultaneous intake with natto as the protein source. Taniguchi et al. confirmed suppression of postprandial glucose elevation after WR intake through the concomitant intake of natto and viscous vegetables and showed that the insulin responses with “WR and natto and viscous vegetables (a high-viscosity mixed meal)” and “WR and boiled soybeans and not viscous vegetables (a low-viscosity mixed meal)” after loading were significantly higher than those with WR. Their results were similar to those of our present study [12]. Additionally, if γ-PGA promotes insulin secretion, the low-γ-PGA natto meal should have lower insulin peaks and higher blood glucose peaks than the high-γ-PGA natto meal, but in fact came out the opposite of that. From these results, it was suggested that γ-PGA did not exert a blood glucose lowering effect through stimulation of insulin secretion, but suppressed the absorption of carbohydrates.

Although the high-γ-PGA natto meal used in the present study had nearly the same carbohydrate and protein content as the low-γ-PGA natto meal, the blood insulin’s IAUC of the high-γ-PGA natto meal in the early phase after eating was lower than that of the low-γ-PGA natto meal. In the study of Taniguchi et al., it was shown that the mean blood insulin’s IAUC of a high-viscosity mixed meal was lower than that of a low-viscosity mixed meal, although the nutritional composition of both test meals was similar, and the meals only differed in their proportion of the water-soluble dietary fiber (viscous substance) in the total dietary fiber [12]. Natto contains a large number of physiologically active substances [20], not only γ-PGA. However, in our study, the difference in the content of γ-PGA might have influenced the difference in the blood insulin’s IAUC between the high- and low-γ-PGA natto meals, because the components other than γ-PGA did not differ between the two test meals. The results of the sensory evaluation of natto in the present study suggested the possibility of inadequate blinding of the participants due to the differences in ”viscosity”, “hardness”, and “flavor” between the high- and low-γ-PGA natto, but we made the ingestion speed of both test meals uniform for all participants. Therefore, we decided that even if participants could distinguish between the two types of natto, it was unlikely to influence the changes in postprandial blood glucose and insulin levels in this study.

This study has some limitations: (1) this was an exploratory study, and the multiplicity of statistical tests was not adjusted; (2) the number of participants and the significant differences in effects between test meals were low. While the data need to be interpreted carefully, this study is the first to indicate the possibility that consumption of high-γ-PGA natto might prevent postprandial hyperglycemia in humans. Based on these results, a confirmatory study should be conducted to evaluate the postprandial anti-diabetic effects of high-γ-PGA natto, including its mechanism.

## 5. Conclusions

Compared to the blood glucose’s IAUC of the high-γ-PGA natto meal 0–120 min after loading, that of WR was significantly higher, whereas that of the low-γ-PGA natto meal showed no difference. Although these values were not adjusted for multiplicity of statistical tests because this study was an exploratory one, the blood glucose’s IAUC of the high-γ-PGA natto meal was the lowest among the three test meals at all time points and was significantly lower than that of low-γ-PGA natto for up to 45 min after the meal. Thus, these results indicate the possibility that concurrent consumption of high-γ-PGA natto may be effective for suppressing an increase in the postprandial blood glucose levels in the early phase after eating in humans.

## Figures and Tables

**Table 1:** Composition and components of each meal.

	WR		Low-γ-PGA Natto Meal		High-γ-PGA Natto Meal	
	Packaged rice	150 g	Packaged rice	150 g	Packaged rice	150 g
			Low-γ-PGA natto	40 g	High-γ-PGA natto	40 g
	Water	150 g	Water	150 g	Water	150 g
**Energy (kcal)**	210		286		288	
**Protein (g)**	2.9		10.2		10.3	
**Fat (g)**	0		3.8		3.8	
**Carbohydrate (g)**	48.5		53.1		53.3	
**γ-PGA (mg)**	0		33.6		269.9	

WR: white rice; γ-PGA: gamma-polyglutamic acid.

**Table 2:** Baseline characteristics of the participants.

Characteristic		Value	
Male/female (number of persons)	12/8		
Age (years)	37.7	±	16.1
BMI (kg/m^2^)	21.4	±	1.7
FPG (mg/dL)	91.3	±	6.4
HbA1c (%)	5.5	±	0.2
Insulin (µU/mL)	5.6	±	2.3
HOMA-IR	1.3	±	0.5
Consumption frequency of natto (number of persons, (%))			
Every day	2	(10.0)	
Almost every day	3	(15.0)	
3–4 times per week	4	(20.0)	
1–2 times per week	6	(30.0)	
Less than once per week	5	(25.0)	

BMI: body mass index; FPG: fasting plasma glucose; HbA1c: hemoglobin A1c; HOMA-IR: homeostasis model assessment of insulin resistance.
